# Human Adaptations to Multiday Saturation on NASA NEEMO

**DOI:** 10.3389/fphys.2020.610000

**Published:** 2021-01-12

**Authors:** Andrew P. Koutnik, Michelle E. Favre, Karina Noboa, Marcos A. Sanchez-Gonzalez, Sara E. Moss, Bishoy Goubran, Csilla Ari, Angela M. Poff, Chris Q. Rogers, Janine M. DeBlasi, Bishoy Samy, Mark Moussa, Jorge M. Serrador, Dominic P. D’Agostino

**Affiliations:** ^1^Human Health, Resilience, & Performance, Institute for Human and Machine Cognition, Pensacola, FL, United States; ^2^Department of Molecular Pharmacology and Physiology, Morsani College of Medicine, University of South Florida, Tampa, FL, United States; ^3^Department of Pharmacology, Physiology and Neuroscience, Rutgers Biomedical and Health Sciences, Newark, NJ, United States; ^4^Division of Clinical & Translational Research, Larkin Health System, Miami, FL, United States; ^5^Department of Psychiatry, Larkin Health System, Miami, FL, United States; ^6^Department of Psychology, Hyperbaric Neuroscience Research Laboratory, University of South Florida, Tampa, FL, United States; ^7^Ketone Technologies LLC, Tampa, FL, United States; ^8^Department of Cardiovascular Electronics, National University of Ireland Galway, Galway, Ireland

**Keywords:** NASA, hyperbaric (underwater), extreme environment, adaptation, saturation

## Abstract

Human adaptation to extreme environments has been explored for over a century to understand human psychology, integrated physiology, comparative pathologies, and exploratory potential. It has been demonstrated that these environments can provide multiple external stimuli and stressors, which are sufficient to disrupt internal homeostasis and induce adaptation processes. Multiday hyperbaric and/or saturated (HBS) environments represent the most understudied of environmental extremes due to inherent experimental, analytical, technical, temporal, and safety limitations. National Aeronautic Space Agency (NASA) Extreme Environment Mission Operation (NEEMO) is a space-flight analog mission conducted within Florida International University’s Aquarius Undersea Research Laboratory (AURL), the only existing operational and habitable undersea saturated environment. To investigate human objective and subjective adaptations to multiday HBS, we evaluated aquanauts living at saturation for 9–10 days via NASA NEEMO 22 and 23, across psychologic, cardiac, respiratory, autonomic, thermic, hemodynamic, sleep, and body composition parameters. We found that aquanauts exposed to saturation over 9–10 days experienced intrapersonal physical and mental burden, sustained good mood and work satisfaction, decreased heart and respiratory rates, increased parasympathetic and reduced sympathetic modulation, lower cerebral blood flow velocity, intact cerebral autoregulation and maintenance of baroreflex functionality, as well as losses in systemic bodyweight and adipose tissue. Together, these findings illustrate novel insights into human adaptation across multiple body systems in response to multiday hyperbaric saturation.

## Introduction

Extreme environments have been explored for over a century to understand human adaptation, extrapolate insights into comparative pathology, and extend human exploration (Tipton, 2016). Salient examples include microgravity, hypobaric, hyperbaric, hypothermic, and hyperthermic environments. These environments provide individual or multiple external stimuli and stressors which are sufficient to disrupt internal homeostasis and induce adaptation processes (Taylor and Cotter, 2006; Tipton, 2016). Adaptations have been observed in subjective psychology and multiple body systems, including cardiovascular, autonomic, musculoskeletal, circadian, pulmonary, endocrine, vestibular, neurosensory, hematological, thermoregulatory, and others (Webb, 1995; Taylor and Cotter, 2006; Kanas and Manzey, 2008; Williams et al., 2009; Pendergast et al., 2015; Tipton, 2016).

Hyperbaric and/or saturation (HBS) environments impact divers (recreation, sport, or professional), personnel operating below sea level (bridge construction, submarine, etc.), and/or patients within pressurized chambers used for hyperbaric oxygen (O_2_) therapy or hyperbaric research. Pendergast et al. (2015) described that these environmental conditions include one or more stimuli including pressure (depth), temperature extremes, and/or an unbreathable ambient medium (Pendergast et al., 2015). Inherent across all HBS environments is the increased partial pressure of inspired gases (P_I_) (Ciarlone et al., 2019). P_I_ is determined by the fractional concentration of gas multiplied by the barometric pressure. At sea level, the barometric pressure is 1 atmosphere absolute (1 ATA/760 mmHg) with 21% O_2_ (0.21 PO_2_) and 78% nitrogen (0.78 PN_2_). Increased sea level depth (+1 ATA per 10 m depth) or forced air compression elevates P_I_, causing O_2_ and/or N_2_ arterial pressure to rise, a physiologic state termed “saturation.” Increased arterial PO_2_ and/or PN_2_ have been demonstrated to increase reactive N_2_ and O_2_ species (RNOS) production, modify redox balance, and impact neurosensory, pulmonary, autonomic, and cardiovascular systems (D’Agostino et al., 2013; Poff et al., 2016; Ari et al., 2019; Ciarlone et al., 2019). Beyond pressure and gas extremes, acute aquatic HBS also influences cardiorespiratory, endocrine, thermoregulatory and renal responses (Pendergast et al., 2015). While much has been learned about acute responses to HBS stressors in controlled laboratory conditions, HBS still remains the most understudied environmental extreme due to inherent experimental, analytical, technical, temporal, and safety limitations (Pendergast et al., 2015; Tipton, 2016), with even less known on the chronic adaptive response in ecological HBS environments as only a small number of historical reports exist in undersea habitats (O’Neal et al., 1965; Pauli and Clapper, 1967; Bayles, 1970; Pauli and Cole, 1970; Anderson and Herrmann, 1971; Nowlis et al., 1972) or pressurized sea level chambers (Seo et al., 1999; Buguet, 2007) at varying depths and lengths of exposure.

Florida International University’s (FIU) Aquarius Undersea Research Laboratory (AURL) is a submerged 400 square feet 74,389 kg habitat 9 km offshore and is considered the world’s only existing underwater research laboratory allowing individuals to live and operate at HBS for up to 3 weeks. The National Aeronautic Space Agency (NASA) Extreme Environment Mission Operation (NEEMO) is a space-flight analog mission conducted within AURL, with similarities to space-flight missions, including mission task load and demands, communication logistics, geographical isolation, extravehicular activities (EVAs), associated risks, and others. Consequently, the NASA NEEMO AURL provides an operational relevant research environment for elucidating the multiday adaption impact of real-world HBS on free-living, highly trained operators (aquanauts: individuals who successfully inhabit AURL for >24 h without resurfacing), allowing for advanced analyses into the multiday adaptive impact of HBS on human psychology and integrated physiology, allowing potential insight into comparative medical pathologies, and/or aiding in the development of mitigative strategies or augmentics to extend human exploration (Smith et al., 2004; Zwart et al., 2009; Strewe et al., 2015; Tipton, 2016; Kim et al., 2018).

**FIGURE 1:**
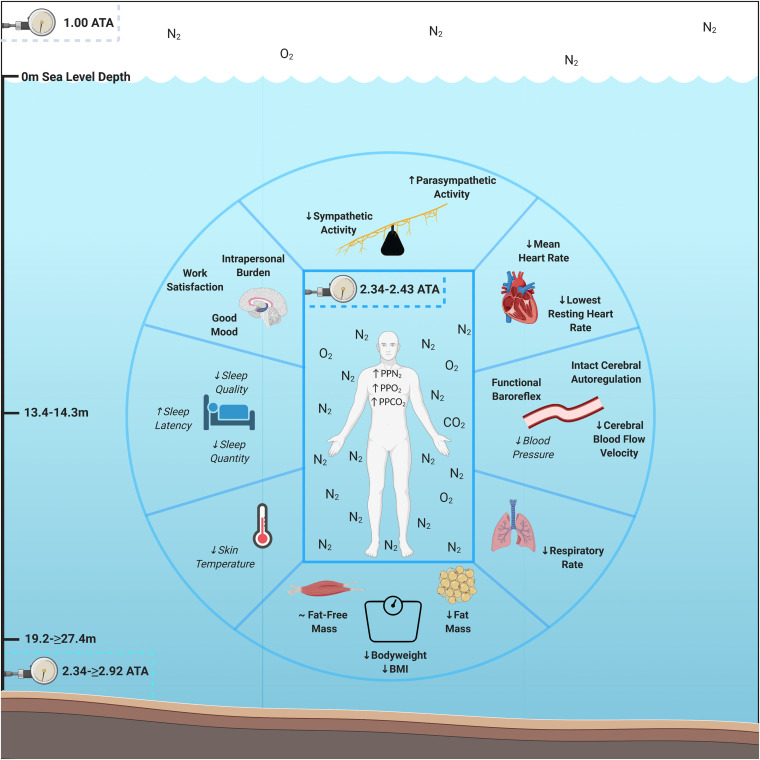
Psychological and physiological adaptations to multiday saturation. Aquanauts exposed to hyperbaric saturation from 2.34 to ≥2.92 atmospheres absolute (ATA) over 9–10 days experienced intrapersonal physical and mental burden, sustained good mood and work satisfaction, decreased heart and respiratory rates, increased parasympathetic and reduced sympathetic modulation, lower cerebral blood flow velocity, intact cerebral autoregulation and maintenance of baroreflex functionality, and losses in systemic bodyweight and adipose tissue. Reductions in skin temperature (5/6 aquanauts) and blood pressure (3/3 aquanauts), as well as elevations in sleep quality (4/6 aquanauts) were observed. Abbreviations: ATA, atmospheres absolute; PP, partial pressure; O_2_, oxygen; N_2_, nitrogen, CO_2_, carbon dioxide; BMI, Body Mass Index. Italicized and un-bolded text indicates non-significance changes.

To investigate human objective and subjective adaptation to multiday HBS, we evaluated each aquanaut’s psychological, cardiac, respiratory, autonomic, thermic, peripheral and central hemodynamics, sleep, and body composition parameters while living at saturation for 9–10 days across NASA NEEMO 22 and 23. We hypothesized that aquanauts would experience psychological burden, reduced mood and work satisfaction, increased heart rate (HR), decrease respiratory rate, increased sympathetic and reduced parasympathetic modulation, elevated skin temperature, hemodynamic dysfunction, reduced sleep quality and quantity, bodyweight and fat-free mass loss, without change in adipose tissue, due to accumulated exposure to AURL HBS multi-stressors. However, we discovered that humans living at HBS for 9–10 days experienced intrapersonal physical and mental burden, sustained good mood and work satisfaction, decreased HR and respiratory rates, increased parasympathetic and reduced sympathetic modulation, lower cerebral blood flow velocity, intact cerebral autoregulation and maintenance of baroreflex functionality, and losses in systemic bodyweight and adipose tissue (Figure 1). These data illustrate multiple novel advancements in understanding human adaption prior to, during, and following multiday HBS in an operationally relevant environmental setting with clear overlap and distinctions between other environmental extremes.

## Materials and Methods

### NASA NEEMO Subject Population, Selection, and Mission Logistics

NASA NEEMO 22 and 23 missions planning began in January 2017 and January 2019, respectively. Eleven aquanauts (male, *n* = 7; female, *n* = 4) composed of astronauts (*n* = 4), research scientists (*n* = 4), and/or professional habitat technicians (*n* = 3) underwent a multistep verification process including, medical and physical evaluation, confirmation of accredited national or international diving certification and experience, and mission training at NASA Johnson Space Center (JSC) to participate in NASA NEEMO 22 and 23. Upon selections, aquanauts underwent continual internet-based planning, training and familiarization along with a week-long mission training and evaluation program at JSC involving equipment, timeline, research projects, and personnel familiarization. 7 days before entering HBS, participants arrived at FIU On-Shore Mission Control Facility in Islamorada, Florida where they underwent pre-mission preparation involving training, planning, dives, etc. JSC and/or ARB training week provided an analogous physical and mental task load environment to within habitat HBS and was used as a normobaric pre-saturation control timepoint. Following 7 days of ARB training, aquanauts dove into the HBS environment (AURL) where they lived and executed full-day mission objectives for 9–10 days which included extravehicular activities, continual communication with FIU On-Shore Mission Control, research implementation, and other activities, analogizing space-flight operational demands and inherent risks. Throughout HBS, aquanauts were supported by Navy Medical Divers and additional support divers to ensure safe mission completion. Within 24 h of resurfacing, aquanauts underwent a 14–18 h desaturation protocol to lower arterial PN_2_. Following resurfacing, participants underwent 2-days of mission debriefing. This period was used for the normobaric post-saturation recovery timepoints. Intrapersonal physical burden, mental burden, mood, work satisfaction, cardiac function, autonomic function, sleep quality, subjective sleep, respiration, and body temperature measurements were taken twice pre-saturation (ARB Pre-Saturation Day 4&5), twice in saturation (Mission Day 4&8), and once post-saturation (Debriefing Day 1). Peripheral and cerebrovascular hemodynamics were measured once pre-saturation (JSC Pre-Saturation), three time during saturation (Mission Day 2 or 3, 5 or 6, 8 or 9), and once post-saturation (Debriefing Day 1). Body composition was measured 24 h prior to saturation and within 24 h of resurfacing. All crewmembers gave informed consent, as approved by the Institute for Human and Machine Cognition, Johns Hopkins University, NASA JSC, and/or European Space Agency Institutional Review Boards. Aquanaut baseline anthropometric characteristics are described in Table 1.

**TABLE 1:** Aquanaut baseline anthropometric characteristics.

**Aquanaut Baseline Anthropometric Characteristics**								
	**Height (cm)**	**Weight (kg)**	**BMI (kg/m^2^)**	**Waist Circumference (cm)**	**Body Fat (%)**	**Fat-Mass (kg)**	**Fat-Free Mass (kg)**	**BMD (g/mL)**
All	177 ± 12	84.7 ± 18.2	26.7 ± 4.0	89.4 ± 13.0	20 ± 7	16.3 ± 4.6	68.4 ± 17.4	1.1 ± 0.0

*Body composition assessment for aquanauts during Pre-Saturation. *N* = 9 (male, *n* = 7; female, *n* = 2). **Data:** Mean ± SD. BMD, Bone Mineral Density; BMI, Body Mass Index.*

### Hyperbaric Saturation and Aquarius Underwater Research Laboratory

Sea-level air (21% O_2_, 78% N_2_, and trace gases) was pumped into AURL via high pressure air compressors from a sea level life support buoy into high pressure gas cylinders. AURL is positioned at 19.2 m sea water depth (2.92 ATA/2219 mmHg) where aquanauts lived and operated primarily at ∼13.4–14.3 m sea water depth (2.34–2.43ATA/1778–1847 mmHg) depending on oceanic tide with intermittent multi-hour EVAs at ≥ 19.2 meters depth (≥2.92 ATA/ ≥ 2219 mmHg). Throughout EVAs, aquanaut buoyancy was set to replicate Mars microgravity and they were monitored for sea water depth to ensure individuals remained within “no decompression” limits under continuous saturated diving conditions. PP_gas_ in each environment was calculated as gas fractional concentration multiplied by total pressure. Corresponding PPO_2_, PPN_2_, and PPCO_2_ are indicated in Table 2. AURL CO_2_ was recorded at 0.3–1.5% surface equivalence value throughout mission, similar to PPCO2 observed within the international space station (Law et al., 2014). Fresh sodasorb and ventilation were used to attenuate further CO_2_ rise.

**TABLE 2:** Aquarius underwater research laboratory hyperbaric environmental parameters.

**Aquarius Underwater Research Laboratory Hyperbaric Environment**			
**Environment**	**Sea Level**	**AURL**	**EVAs**

Timeline	Pre-Saturation and Post-Saturation	Saturation	Saturation
Exposure	–	9–10 days	∼2–6 h
Sea Water Depth	0 m	13.4–14.3 m	≥19.2 m
Total Pressure	1 ATA/760 mmHg	2.34-2.43 ATA/1778-1847 mmHg	≥2.92 ATA/ ≥2219 mmHg
PPO_2_	0.21 ATA/160 mmHg	0.49-0.51 ATA/372-388 mmHg	≥0.61 ATA/≥464 mmHg
PPN_2_	0.78 ATA/593 mmHg	1.83-1.90 ATA/1391-1444 mmHg	≥2.28 ATA/≥1733 mmHg
PPCO_2_	0.0004 ATA/0.3 mmHg	0.006–0.015 ATA/5-11 mmHg	–

*Environment and corresponding exposure, timeline and gas pressures are indicated. AURL, Aquarius Underwater Research Laboratory; EVAs, Extra-Vehicular Activities; ATA, Atmospheric absolutes/barometric pressure; mmHg, millimeters of mercury; PP, partial pressure; O2, oxygen; N2, nitrogen; CO2, carbon dioxide.*

### Intrapersonal Physical Burden, Mental Burden, Mood and Work Satisfaction

Aquanauts completed a multi-question intrapersonal mental burden, physical burden, mood, and work satisfaction survey to evaluate the busy/hectic, physically strenuous, mentally taxing, post-mission day fatigue, tenseness, and stiffness, headache and pain using a 1–10 Likert scale at different timepoints, always at the end of each day as previously described (O’Griofa et al., 2013). Intensity scores were separated into five categories: 1–2, 3–4, 5–6, 7–8, and 9–10 which were interpreted as none, very mild, mild, moderate, and severe, respectively. Cumulative mission impact on subjective quality metrics of mood and work satisfaction. Quality scores were separated into five categories: 1–2, 3–4, 5–6, 7–8, and 9–10 which were interpreted as very poor, poor, fair, good, and excellent, respectively. Eight subjects (male, *n* = 6; female, *n* = 2) complied with all timepoints and were included in analyses.

### Cardiac and Autonomic Function

Aquanauts cardiovascular and autonomic function were measured throughout rest. Polar V800 (Polar, Kempele, Finland) Watch and H7 Chest Strap technology was utilized to gather electrocardiographic signals (R–R intervals) across rest and activity (Giles et al., 2016; Caminal et al., 2018). Briefly, prior to bed, subjects dampened the H7 chest strap, affixed the chest strap inferior to pectoral muscles and superficial to xyphoid process, verified recording initiation via V800 (HR) display, underwent troubleshooting procedures if continuous HR was not accurately displayed, wore both the watch and chest strap throughout the duration of sleep, and stopped recording upon cessation of sleep. To ensure that subjects wore devices on designated days, internal measurement dates were validated. Cardiovascular and autonomic function were analyzed from R–R intervals using Kubios 3.3.1 (Kubios Oy, Kuopio, Finland) software. Heart rate variability parameters were measured, analyzed, and interpreted per consensus guideline (Malik, 1996) as previously described by our group (Sanchez-Gonzalez and Figueroa, 2013). All R–R intervals were manually inspected for artifacts, premature beats and non-sinus tachycardia episodes by a trained researcher for the entire sampling period. Artifacts were then manually removed via interpolation excluding non-analyzable and non-reliable raw data. First hour of recording therefore was eliminated uniformly across all samples to account for sleep latency and to minimize artifacts related to physical movement and missing data due to malfunctioning devices. A sample size of 4 h was used regardless of sleep onset and phases to reflect sleep cycles and in a similar approach to previous studies (Finley and Nugent, 1995; Goto et al., 1997; Herzig et al., 2017; Jawabri and Raja, 2020), Kubios artifact correction algorithm with medium threshold was utilized to remove artifacted waveforms automatically (Gisselman et al., 2020), R–R time series interpolation rate of 4 HZ was used. The HRV frequency bands utilized were very low frequency (VLF) (0–0.04 Hz), low frequency (LF) (0.04–0.15 Hz), and high frequency (HF) (0.15–0.4 Hz). The spectrum for the selected R–R interval sample was calculated with Welch’s periodogram method (FFT spectrum), 300 S window width, and window overlap 50% (Li et al., 2019). Seven subjects (male, *n* = 5; female, *n* = 2) complied with all timepoint measurements, did not obtain errors in data collection, and were included in analyses.

### Peripheral and Cerebrovascular Hemodynamics

Aquanauts were assessed during waking hours to determine beat-by-beat middle cerebral artery flow velocity (MCAv), systolic blood pressure (SBP), diastolic blood pressure (DBP), mean arterial pressure (MAP), and estimated HR. Transcranial Doppler measurements were obtained at the *trans*-temporal window with a 2 MHz probe (DWL, Germany) as previously described (Aaslid et al., 1982). The Doppler probe was initially fitted for each aquanaut during JSC pre-saturation. The angle of the probe was glued in place using a custom-made probe holder and was attached via custom-made Velcro headbands. Beat-by-beat blood pressure was determined by finger plethysmography (Portapres, Finapres Medical Systems, Netherlands) on the non-dominant hand. A height correction unit (Finapres Medical Systems, Netherlands) was worn to correct for blood pressure measurements. Subjects completed baseline standing measurements, and a prone-to-stand protocol. During the prone-to-stand protocol, aquanauts began lying prone 2 min, followed by standing for 1 min. Aquanauts repeated this protocol at least once. Any stands with dropouts in data were removed, and the remaining stands were averaged for each mission day. The change in MCAv, MAP, and estimated HR between prone and standing conditions were computed from the averaged values in the 25-s sections immediately before and 30 s after the aquanauts stood up. The drop in MCAv and MAP during the stand were determined as well as measures of cerebral autoregulation by measuring autoregulatory index and the time it took to reach the lowest MCAv value (nadir). Autoregulatory index was used to assess cerebral autoregulation based on Tiecks’ predicted curve fit modeling method (Tiecks et al., 1995), which uses a mathematical model calculating theoretical cerebral blood flow responses to the given blood pressure input. The lowest mean square error between the actual and theoretical MCAv yields the curve of best fit on a scale from 0 to 9, 0 indicating no autoregulation when MCAv passively changes with MAP, and 9 indicating the best observed autoregulatory response when MCAv remains relatively constant despite a change in MAP. Data were acquired using DATAQ acquisition software with a sampling rate of 1,000 Hz. Both MCAv and blood pressure were visually inspected, and any artifacts or signal loss identified were removed and linearly interpolated using a custom-written MATLAB script (MathWorks, Natick, MA, United States) prior to analyses. Static blood pressure was obtained at the brachial artery using a sphygmomanometer and stethoscope. From six male participating subjects, four aquanauts complied with all timepoint measurements during baseline standing conditions (male, *n* = 4) and three completed the prone-to-stand protocol (male, *n* = 3).

### Sleep Quality, Respiration and Body Temperature

Aquanauts were evaluated for sleep quantity, quality, respiration and skin temperature throughout rest (sleep) using OURA which is a 15 g multisensory technology (de Zambotti et al., 2017; Maijala et al., 2019). This technology uses infrared light and a photosensor to gather beat-by-beat blood volume pulse architecture on the dominant ring finger palmar artery. 3D accelerometer gathered body movement amplitude and intensity with 30 s frequency resolution. Negative temperature coefficient thermistor gathered finger skin temperature with a 60 s and 0.07°C resolution. Sleep–wake patterns and characteristics were derived from combination of pulse rate, features from bb intervals, pulse amplitude, and motions obtained via standard actigraphy (de Zambotti et al., 2017). OURA devices were pre-fitted for dominant hand ring finger at JSC pre-mission training week to ensure individualized ring size fit. To ensure aquanauts wore devices on designated days, internal device dates were validated. Total sleep (hours), rapid eye movement (REM; hours), deep (hours), REM/total, deep/total, latency to sleep (minutes), average HR variability (Root Mean Square of Successive Differences, RMSSD; ms), average HR (beats per minute), lowest rest HR (beats per minutes), respiratory rate (breaths per minutes), and skin temperature (Celsius; °C) were gathered. Six aquanauts were instructed to wear the device on the same hand and finger throughout all measurements with 100% compliance across timepoints. All obtained data were included in analyses (male, *n* = 6).

### Subjective Sleep Assessment

Immediately before and upon completion of sleep, participants completed a modified Pittsburg Sleep Diary adapted to evaluate subjective sleep pre-, during, and post-saturation for trouble falling asleep, sleep quality, sleeplessness, pre-mission day fatigue, and alertness in extreme environmental conditions (Griofa et al., 2011), using a 1–10 Likert scale. Intensity scores were separated into five categories: 1–2, 3–4, 5–6, 7–8, and 9–10 were interpreted as none, very mild, mild, moderate, and severe, respectively. Number of Sleep Disturbances were quantified upon waking. Seven subjects (male, *n* = 5; female, *n* = 2) complied with all timepoints and were included in analyses.

### Body Composition

Aquanauts underwent anthropometric assessment to determine both systemic and region-specific alterations in tissue composition. Subject daily routines were pre-assigned throughout. Anthropometric data were gathered across the same environment and routine at similar daily timepoints (10:00–14:00) which illustrated overlapping pre-saturation and post-saturation subject timepoints to control for lifestyle-induced variability. Anatomical landmarks were indicated with a marking pen to ensure measurements were gathered at the same anatomical sites. Weight (±0.1 kg) and height (nearest cm) were gathered using a digital scale and stadiometer, respectively. The dominant extremity was used for gathering remaining anthropometric data. Waist circumference was measured between the right and left iliac crest using standard measuring tape. Leg circumference was measured halfway between the greater trochanter and patella. Arm circumference was gathered between the lateral humerus greater tuberosity and olecranon. Three-point sex-specific caliper skinfolds (male: mid-chest, mid-thigh, abdominal; female: mid-upper arm, mid-thigh, suprailiac; Schmidt and Carter, 1990) were evaluated as previously described (Jackson and Pollock, 1985). A-mode ultrasound muscle thickness (chest and mid-thigh; mm; BodyMetrix Pro, Brentwood, CA, United States) were gathered across corresponding skinfold regions (Utter and Hager, 2008; Wagner, 2013). All scans where performed with the transducer held perpendicular to the skin and starting at the visible lateral muscular border and finishing at the visible medial muscular border. All measurements were performed twice. If measurements were no within 0.1 kg, 1 cm, 5, 2, and 2 mm across weight, height, circumference, skinfold, and ultrasound, respectively, a third was taken. Values were averaged across timepoints. Body mass index (BMI; kg/m^2^), body fat (%), fat-mass (kg), fat-free mass (kg), and bone density (g/mL) were calculated from anthropometric data (Jackson and Pollock, 1985). Nine subjects (male, *n* = 7 and female, *n* = 2) were available within 24 h post-resurfacing for body composition pre- to post-analyses. Of the nine subjects, six subjects gathered multiple muscle thickness measurements and were included in analyses (male, *n* = 6).

### Statistical Analyses

GraphPad Prism 8 software was used for all statistical analyses. Within subject parameters were averaged within timepoint (Pre-Saturation, Saturation, Post-Saturation). The Shapiro–Wilk test was performed to determine normal distribution. Logarithmic transformation was performed for all parameters determined to not be normally distributed. Paired *t*-tests were performed for the comparison of two timepoints (Pre-Saturation versus Post-Saturation). Repeated Measures One-Way ANOVA followed by Tukey’s *post hoc* was used for >3 comparisons and Fisher LSD *post hoc* for ≤3 comparisons to control for family wise error. Pearson correlation analyses were conducted where overlapping timepoints and measurements were reported between OURA and Polar devices (Mean HR and RMSSD). Significance is indicated where *p* < 0.05. Data are reported as mean ± SD.

## Results

### Intrapersonal Physical Burden, Mental Burden, Mood and Work Satisfaction

**FIGURE 2:**
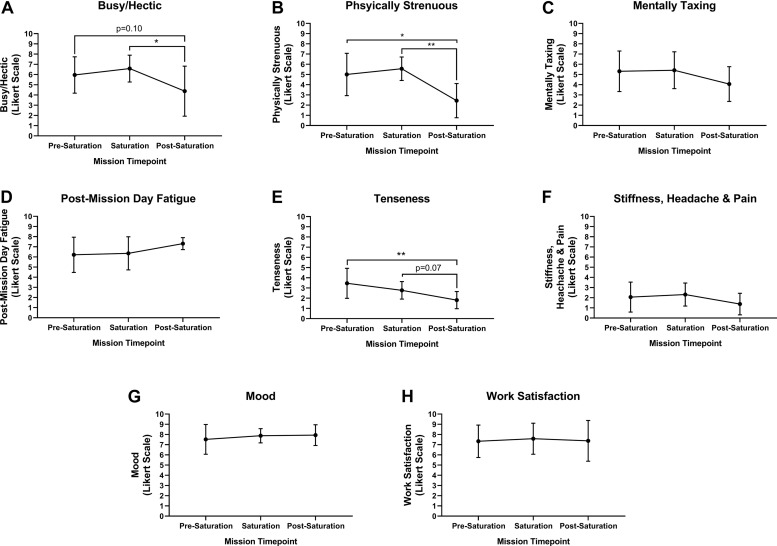
Intrapersonal burden, mood and work satisfaction. Subjective assessment of **(A)** Busy/Hectic, **(B)** Physical Strenuous, **(C)** Mentally Taxing, **(D)** Post-Mission Day Fatigue, **(E)** Tenseness, **(F)** Stiffness, Headaches and Pain were assessed using Likert Scaling to determine intrapersonal physical and mental burden. Subjective assessment of **(G)** Mood and **(H)** Work Satisfaction were quantified using Likert Scaling. *N* = 8 (male, *n* = 6; female, *n* = 2). **Data:** Mean ± SD. *p < 0.05, **p < 0.01. Raw *p*-values are reported for non-significant changes where *p* ≤ 0.10.

Aquanauts reported mild and moderate levels of business/hecticness at pre-saturation and saturation, respective. However, business/hecticness was significantly reduced from saturation to very mild post-saturation (Figure 2A; *p* = 0.026). Physical strenuousness was mild at pre-saturation and saturation but reduced significantly to none post-saturation compared to pre-saturation (*p* = 0.012) and saturation (Figure 2B; *p* = 0.004). Mental taxation was mild at pre-saturation and saturation, and very mild post-saturation, but was not significantly different across timepoints (Figure 2C). Post-mission day fatigue was mild at pre-saturation and saturation, but moderate post-saturation, but was not significantly different across timepoints (Figure 2D). Tenseness was very mild at pre-saturation and saturation, but significantly decreased to none post-saturation compared to other timepoints (Figure 2E; *p* = 0.005). No stiffness, headaches, and pain were indicated across timepoints (Figure 2F). Mood and work satisfaction were good throughout all timepoints (Figures 2G,H).

### Cardiorespiratory, Autonomic and Temperature Regulation

**FIGURE 3:**
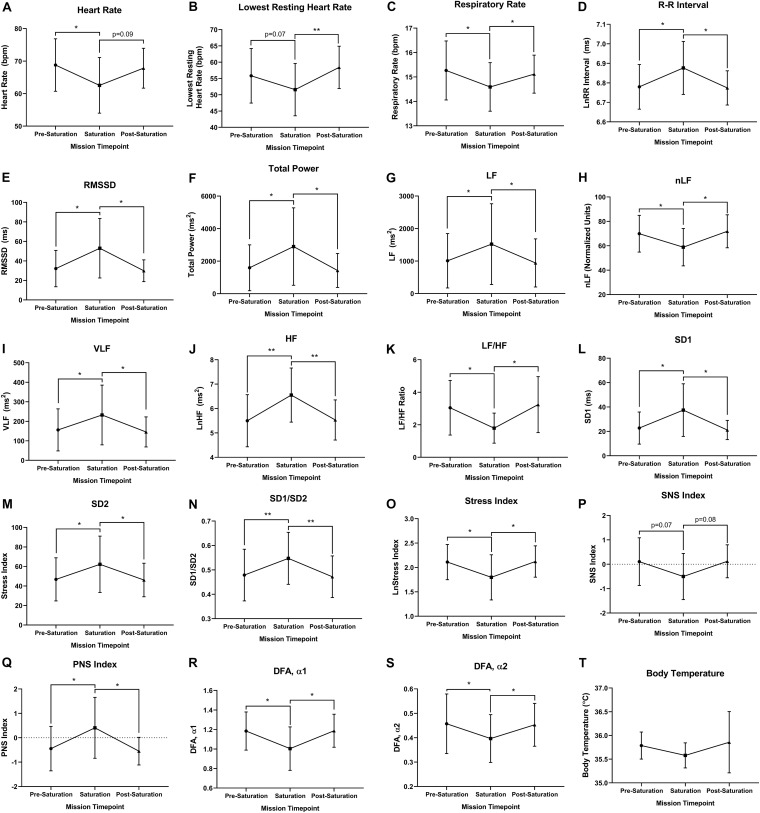
Cardiorespiratory, autonomic, and thermal regulation. **(A)** Mean and **(B)** lowest heart rate were quantified via electrocardiography at rest. **(C)** Respiratory rate was algorithmically determined via beat-by-beat R–R intervals at rest. **(D)** Autonomic time domain mean R–R intervals and **(E)** mean Root Mean Square of Successive Differences (RMSSD) were derived from electrocardiography R to R time intervals at rest. R–R intervals were not normally distributed and logarithmically transformed lnRR. **(F)** Autonomic frequency domain mean Total power, **(G)** very low frequency (VLF; <0.04 Hz), **(H)** low frequency (LF; 0.04–0.15 Hz), **(I)** normalized low frequency [nLF; nLF = (LF/(Total Power-VLF)], **(J)** high frequency (HF; 0.15–0.4 Hz), and **(K)** ratio of low frequency to high frequency (LF/HF) were all quantified from resting electrocardiography R to R time intervals at rest. **(L)** Poincaré perpendicular (SD1), **(M)** parallel (SD2) standard deviation, and **(N)** SD1/SD2 were determined via non-linear assessment of heart rate variability by quantitative two-dimensional vector analysis of a Poincaré plot at rest. **(O)** Short- (DFAα1) and **(P)** long-term detrended fluctuation analysis (DFAα2) used to analyze non-stationary systems at rest. **(Q)** Stress, **(R)** sympathetic nervous system (SNS), and **(S)** parasympathetic nervous system (PNS) were analyzed as composite metrics of each respective arm of autonomic regulation at rest. **(T)** Body temperature was gathered via skin thermistor during rest. **(A,D–S)** *N* = 7 (male, *n* = 5; female, *n* = 2). **(B,C,T)** *N* = 6 (male, *n* = 6) **Data:** Mean ± SD. RMSSD, Root Mean Square of Successive Differences; VLF, very low frequency; LF, low frequency; nLF, normalized low frequency; HF, high frequency; SD1, Poincaré Perpendicular Standard Deviation; SD2, Poincaré Parallel Standard Deviation; DFAα1, short-term detrended fluctuation analysis; DFAα2, long-term detrended fluctuation analysis; SNS, sympathetic nervous system; PNS, parasympathetic nervous system. *p < 0.05, **p < 0.01. Raw *p*-values are reported for non-significant changes where *p* ≤ 0.10.

Heart rate was significantly lower during saturation compared to pre-saturation (*p* = 0.049), while trending lower from during saturation compared to post-saturation (Figure 3A; *p* = 0.089). Lowest resting HR was trending lower during saturation compared to pre-saturation (*p* = 0.069), while significantly lower during saturation compared to post-saturation (Figure 3B; *p* = 0.008). Respiratory rate was significantly lower in saturation compared to pre- (*p* = 0.012) and post-saturation (Figure 3C; *p* = 0.039). R–R Interval, RMSSD, Total Power, VLF, LF, HF, Poincaré Perpendicular Standard Deviation (SD1), Poincaré Parallel Standard Deviation (SD2), SD1/SD2, and PNS index were significantly higher during saturation compared to either pre- or post-saturation timepoints (Figures 3D–H,J,L–N,S; *p* < 0.050). Alternatively, nLF, LF/HF, Short-Term Detrended Fluctuation Analysis (DFAα1), Long-Term Detrended Fluctuation Analysis (DFAα2), and Stress Index were all significantly lower during saturation compared to pre- and post-saturation timepoints (Figures 3I–K,O–Q; *p* < 0.050). SNS Index showed trends for reductions during saturation compared to pre- (*p* = 0.077) and post-saturation timepoints (Figure 3R; *p* = 0.071). While 5/6 aquanauts observed a reduction in skin temperature during saturation, this was not significant across timepoints (Figure 3T).

### Peripheral and Cerebrovascular Hemodynamics

**FIGURE 4:**
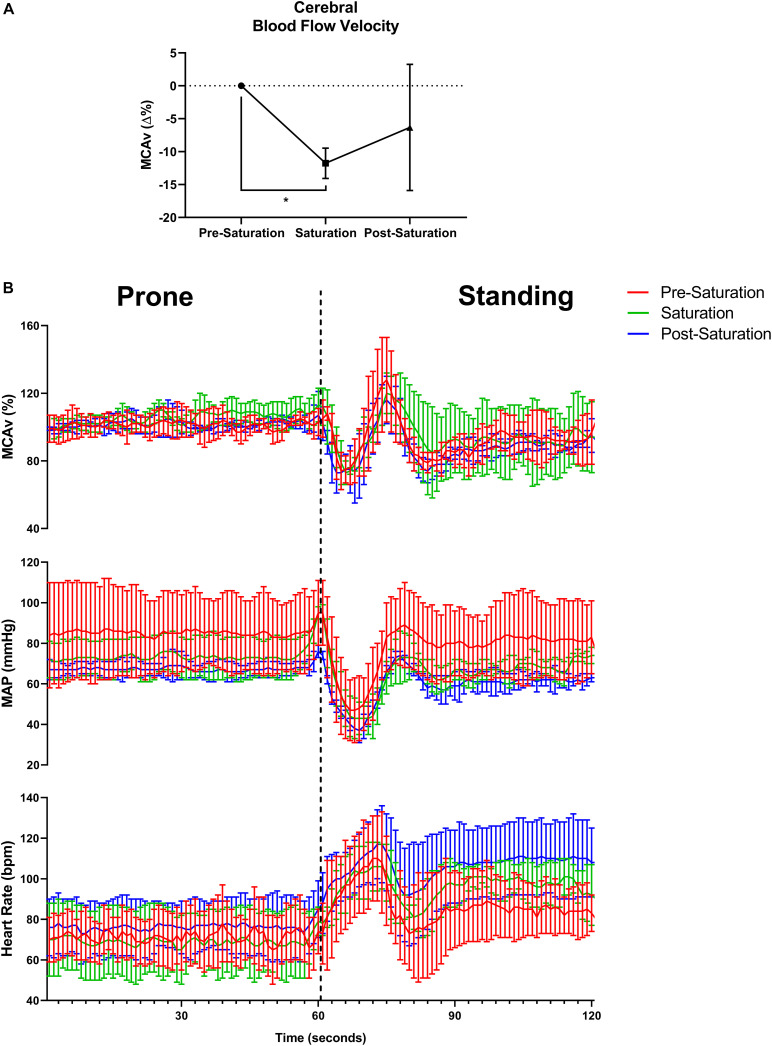
Baseline cerebral blood flow velocity, cerebral autoregulatory and baroreflex responses. **(A)** Percent change in baseline cerebral blood flow velocity from pre-saturation measured at the middle cerebral artery (MCAv%) across timepoints. *N* = *4* (male, *n* = 4). **(B)** One-second averages of beat-by-beat MCAv, mean arterial pressure (MAP), and estimated heart rate derived from systolic blood pressure peaks during waking prone (lying) position into standing position capturing the cerebral autoregulatory and baroreflex responses. *N* = 3 (male, *n* = 3). **Data:** Mean ± SD. MCAv%, middle cerebral artery velocity percent change; MAP, mean arterial pressure. *p < 0.05.

Baseline middle cerebral artery velocity (MCAv) when standing significantly decreased 11.8% during saturation compared to pre-saturation (*p* = 0.017, Figure 4A). The peripheral and cerebrovascular responses during the prone-to-stand protocol are illustrated in Figure 4B and Table 3. Despite no significant differences across timepoints in any variable when prone, there was an overall reduction in blood pressure (MAP and SBP) measures during saturation in 3/3 aquanauts. When assessing the change from prone when in steady-state standing conditions 30 s after the aquanauts stood up, estimated HR (ΔHR) was significantly elevated in saturation compared to pre-saturation (*p* = 0.005) with similarly elevated, but more variable ΔHR in post-saturation (*p* = 0.056). There were no significant differences in ΔMAP or ΔMCAv when standing, but there was a 16% reduction in MCAv during saturation compared to only 8% reduction during pre-saturation. There were no significant differences in cerebral autoregulatory index when assessing cerebral autoregulatory response during transition from prone to standing. There were also no differences in the magnitude of the drop in MAP or MCAv across timepoints.

**TABLE 3:** Aquanaut waking peripheral and cerebrovascular hemodynamic parameters.

**Aquanaut Waking Peripheral and Cerebrovascular Hemodynamics**				
	**Pre-Saturation**	**Saturation**	**Post-Saturation**	***P-*value**

**Prone**				
MAP, mmHg	85.4 ± 18.6	73.2 ± 10.2	67.3 ± 3.7	*0.125*
SBP, mmHg	128.5 ± 24.2	107.5 ± 20.1	95.0 ± 10.8	*0.150*
DBP, mmHg	66.9 ± 18.4	57.3 ± 6.0	53.9 ± 2.1	0.217*
HR, bpm	73.0 ± 14.0	69.3 ± 15.7	77.0 ± 13.9	*0.369*
MCAv, cm/s	47.9 ± 7.3	43.6 ± 5.3	44.5 ± 8.6	*0.224*
**Static Change from Prone when Standing (30 s After Stand)**				
ΔMAP, mmHg	−3.7 ± 2.9	−5.6 ± 7.3	−5.5 ± 7.4	*0.805*
ΔHR, bpm	12.8 ± 8.0	30.1 ± 9.5	32.3 ± 16.3	*0.012*
ΔMCAv, %	−8.4 ± 6.5	−16.1 ± 21.5	−12.8 ± 3.1	0.475**
**Prone-to-Stand Cerebral Autoregulatory Response (During Stand)**				
Cerebral Autoregulatory Index	4.5 ± 0.9	3.9 ± 0.5	3.7 ± 0.8	0.434*
ΔMAP, mmHg	−39.0 ± 3.9	−35.4 ± 7.2	−31.7 ± 9.4	*0.117*
ΔMCAv, %	−31.8 ± 8.1	−38.7 ± 2.1	−40.7 ± 13.9	*0.624*
Time to MAP nadir, s	7.5 ± 1.2	8.3 ± 1.2	7.3 ± 0.5	*0.275*
Time to MCAv nadir, s	4.4 ± 1.2	5.5 ± 1.7	4.2 ± 3.0	*0.272*

Waking heart rate, blood pressure, and cerebral blood flow velocity values across pre-saturation, saturation, and post-saturation timepoints during the prone-to-stand protocol. Prone values are the average of the 25 s window before the stand. The static change from prone when standing is the difference between values before the stand and 30 s after the stand. The prone-to-stand autoregulatory response reflects the drop in blood pressure and cerebral flow velocity during the stand and the time it took to reach the lowest values (nadirs). *N* = 3 (male, *n* = 3). **Data:** Mean ± SD. MAP, mean arterial pressure; SBP, systolic blood pressure; DBP, diastolic blood pressure; HR, heart rate; MCAv, middle cerebral artery flow velocity. *Values were logarithmically transformed prior to analyses due to non-normal distribution; **values had a positive constant value [34.1; “33.1” (maximum negative number value multiplied by −1) plus “1”; original value + “33.1 + 1 = 34.1] added prior to logarithmic transformation prior to analyses due to negative values and non-normal distribution. *ns, p* > *0.05* across comparisons.

### Sleep Quantity and Quality

#### Objective Sleep

**FIGURE 5:**
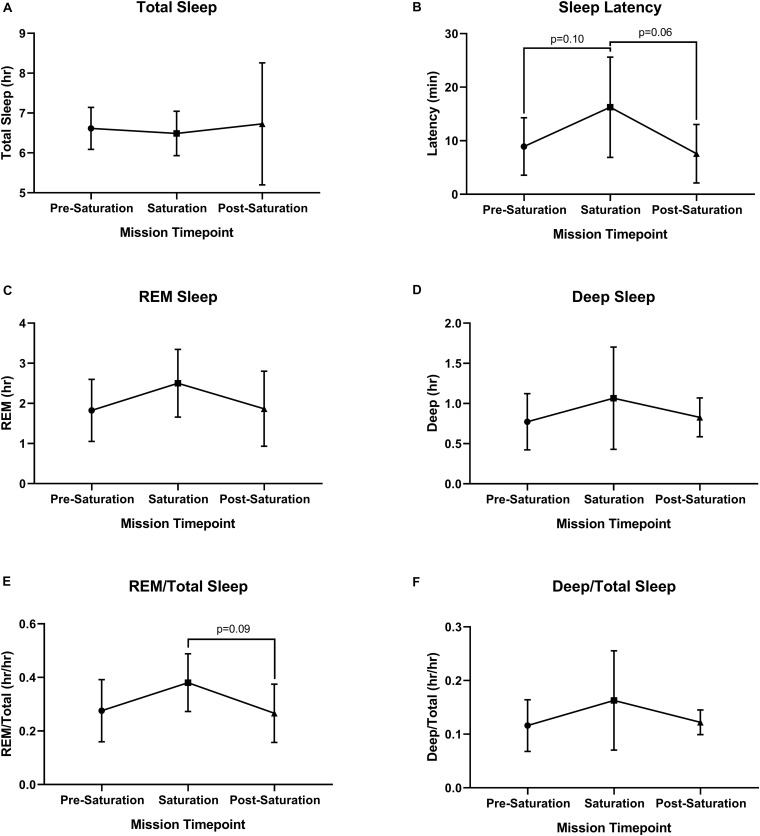
Objective sleep quantity and quality. **(A)** Total, **(B)** Latency, **(C)** Rapid Eye Movement (REM), **(D)** Deep, **(E)** ratio of REM to Total Sleep (REM/Total), and **(F)** ratio of Deep to Total Sleep (Deep/Total) were assessed during rest across timepoints. *N* = 6 (male, *n* = 6) **Data:** Mean ± SD. REM, rapid eye movement. Raw *p*-values are reported for non-significant changes where *p* ≤ 0.10.

No significant differences were found across sleep parameters between the timepoints (Figures 5A–F). However, sleep latency (*p* = 0.061) and REM/total (*p* = 0.093) trended higher during saturation, compared to pre-saturation and post-saturation timepoints. 4/6 aquanauts experienced reduced total sleep along with increased REM and deep/total sleep.

#### Subjective Sleep

**FIGURE 6:**
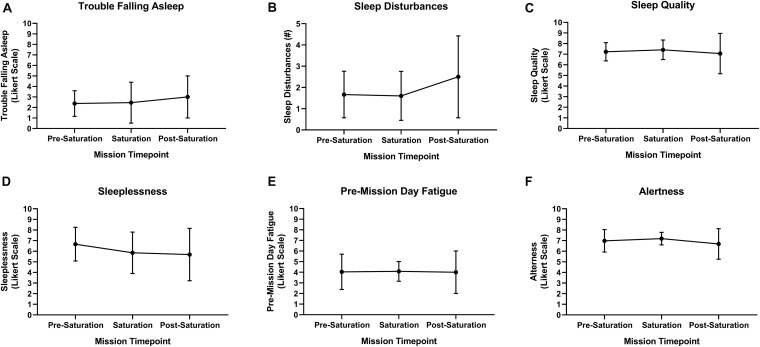
Subject sleep quality. **(A)** Trouble falling asleep, **(B)** sleep disturbances, **(C)** sleep quality**, (D)** sleeplessness**, (E)** pre-mission day fatigue, and **(F)** alertness were subjectively assessed upon waking via sleep diary. *N* = 8 (male, *n* = 6; female, *n* = 2). **Data:** Mean ± SD.

Aquanauts reported no trouble falling asleep at pre-saturation and saturation, and very mild trouble falling asleep post-saturation (Figure 6A). Total number of sleep disturbances was 1.7, 1.6, and 2.5 at pre-saturation, saturation, and post-saturation timepoints, respectively (Figure 6B). Sleep quality and alertness were moderate throughout all timepoints (Figures 6C,F). Moderate sleeplessness was reported pre-saturation (Figure 6D). Mild sleeplessness was reported during and post-saturation. Very mild pre-mission day fatigue was reported throughout. No significant changes were observed across all subjective sleep parameters (Figure 6).

### Body Composition

**FIGURE 7:**
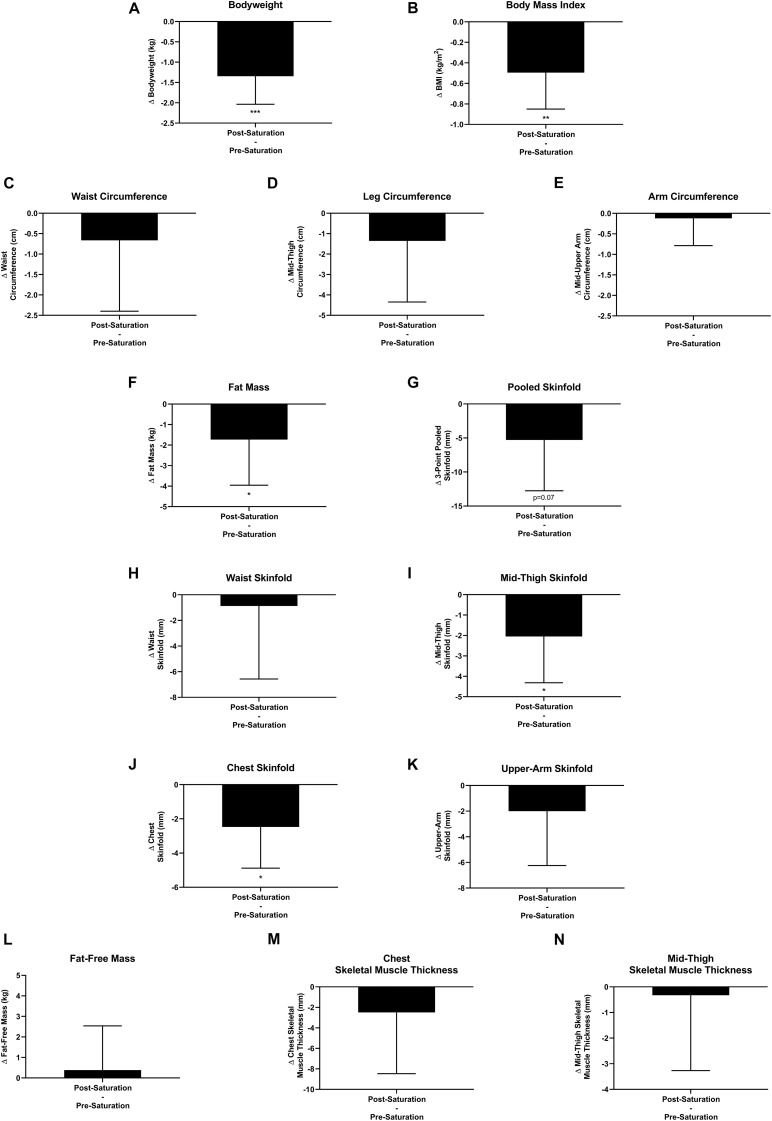
Body composition. Changes (Δ) in **(A)** body weight, **(B)** body mass index (BMI), and **(C–E)** multipoint circumference measurements, **(F)** fat mass, **(G–K)** multipoint skinfold, **(L)** fat-free mass, and **(M,N)** muscle thickness measurements were assessed pre-saturation and post-saturation. **Data:** Mean ± SD. **(A–L)** *N* = 9 (male, *n* = 7; female, *n* = 2). **(M,N)** *N* = 6 (male, *n* = 6). BMI, Body Mass Index. ^∗^*p* < 0.05, ^∗∗^*p* < 0.01, ^∗∗∗^*p* < 0.001. Raw *p*-values are reported for non-significant changes where *p* ≤ 0.10.

Aquanauts had significant reductions in bodyweight (−1.3 ± 0.7 kg; *p* = 0.0004), BMI (−0.5 ± 0.4 kg/m^2^; *p* = 0.003), fat mass (−1.7 ± 2.2 kg; *p* = 0.048), mid-thigh skinfold (−2.0 ± 2.3 mm; *p* = 0.027), and chest skinfold (−2.5 ± 2.4 mm; *p* = 0.036; Figures 7A,B,F,I,J). While all remaining peripheral and central circumference and skinfold measurements reduced post-saturation, they were not significantly altered (Figures 7C–E,G,H,K). Fat-free mass and muscle thickness measurements were not significantly altered (Figures 7L–N).

## Discussion

Research into the HBS environmental adaptation response has been largely reliant on the acute setting (<24 h) due to well characterized difficulties and limitations assessing psychological and physiological responses in aqueous and pressurized gas mediums (O’Neal et al., 1965; Pauli and Clapper, 1967; Bayles, 1970; Pauli and Cole, 1970; Anderson and Herrmann, 1971; Nowlis et al., 1972; Buguet, 2007; Tipton, 2016). We found that highly trained aquanaut operators exposed to HBS over 9–10 days in the world’s only undersea laboratory (Smith et al., 2004; Zwart et al., 2009; Strewe et al., 2015; Kim et al., 2018) experienced intrapersonal physical and mental burden, sustained good mood and work satisfaction, decreased HR and respiratory rates, increased parasympathetic and reduced sympathetic modulation, lower cerebral blood flow velocity, intact cerebral autoregulation and maintenance of baroreflex functionality, and losses in systemic bodyweight and adipose tissue. These findings illustrate multiple novel insights into the unique human adaptation in response to multiday HBS. These findings also illustrate that NASA NEEMO multiday HBS acts as an analog to multiday spaceflight via similar highly trained operational population, inherent mission risks and demands, daily task schedule, confined space, psychological stressors, delayed communication medium, EVAs with matched buoyancy and weightlessness, elevated gas exposures, reduced sleep quantity, increased sleep latency, and reduced bodyweight and fat-free mass changes. However, NASA NEEMO HBS is dissimilar to spaceflight as it results in higher pressurized gas exposures, non-continuous microgravity only during EVAs, intact hemodynamics and baroreflex, increased parasympathetic modulation, reduced sympathetic modulation, and adipose tissue atrophy.

### Intrapersonal Physical Burden, Mental Burden, Mood and Work Satisfaction

We report that aquanauts experienced intrapersonal and/or mental burden during pre-saturation and saturation timepoints which lowered post-saturation, except for post-mission day fatigue which remained elevated throughout. This psychological assessment confirms the stress experienced during pre-saturation and saturation, in agreement with prior studies in multiday HBS (Bayles, 1970; Nowlis et al., 1972) and extreme environments, such as space flight and microgravity (Kanas and Manzey, 2008; Barrett and Martin, 2014). Interestingly, while intrapersonal physical and mental burden were moderately high pre-saturation and post-saturation, mood and work satisfaction remained high throughout, paralleling short duration (13 days) spaceflight and bed rest (15 days) evaluations where astronauts and participants were able to maintain high positive mood and work satisfaction regardless of mission or study stressors, respectively (Lichao et al., 2014; Liu et al., 2016). Interestingly, socially isolating environments in combination with sleep deprivation for as short as 72 h has been shown to increase metrics of fatigue and negatively influence psychology and mood. Thus, the observed maintenance of good mood and work satisfaction in aquanauts, even with elevated fatigue, may be attributable, at least in part, to altered sleep quality. This bidirectional influence of psychological and physiological parameters is well established (Biddle, 2008), and has also been elucidated in space flight and analogs, as positive mood was associated with adaptive catecholamine and neurotransmitter response during a 500-day space flight simulation (Wang et al., 2014; Bartone et al., 2018). Unique to AURL is the saturated environment with elevated gas pressures and resulting increased circulating gas PP’s (Table 2). While subjects did not reach hyperoxia based on the Undersea Hyperbaric Medical Society classification of 1.4ATA at 100% O2 (1.4 PPO2), subjects were still exposed to elevated levels over a 9 or 10-day period, > 2.34x the typical amount of O_2_ and N_2_ inhaled. While not assessed in the present analyses, one of the few reports in multiday HBS confirmed the elevations in inflammatory and nitrogen/oxidative stress (RNOS) in this setting (Smith et al., 2004; Strewe et al., 2015). This is in line with the predicted consequences from elevated exposure to O_2_ likely uncoupled to cellular respiration and consequently shuttled to RNOS generation, commonly reported in the HBS environment (Dean and D’Agostino, 2008; Ciarlone et al., 2019). While oxidative stress has not been causally linked to psychological changes, it is associated with a number of psychological disorders and is hypothesized to be a facilitative aspect of psychological adaptations (Salim, 2014). Additionally, elevated PN_2_ and PCO_2_ have psychological impacts (Kirkland and Cooper, 2019; Patel et al., 2020). Interestingly, we did not observe any subjective elevations in intrapersonal physical burden, mental burden, mood, or work satisfaction within the saturated environment compared to pre-saturation. However, we cannot exclude that pre-selection of “psychologically fit,” highly trained populations (aquanauts) who may be uniquely equipped with “psychological counter measures,” may have influenced these subjective outcomes. Prior studies have found qualified and pre-selected operators were able to mitigate behavior and performance declines in an extreme environment for a similar duration (≤14 days) (Palinkas, 2001; Bartone et al., 2018). None the less, these results serve to confirm the intrapersonal physical and mental burden of NASA NEEMO training and execution before and after HBS, even though mood and work satisfaction remained good throughout, in accordance with prior HBS and other environmental extremes.

### Cardiac, Respiratory, Autonomic, Thermic, and Hemodynamic Regulation

We observed that resting HR and respiratory rate were significantly depressed within multiday HBS. Lower HR and respiratory rate are both in line with reports in acute HBS environments (Adamiec, 1977; Goyal et al., 2020). While HR and respiration have been historically evaluated in multiday HBS using wrist pulse measurements, they were never compared across controlled timepoints or using overlapping objective analytic techniques, limiting interpretation and understanding in adaptive response (Bayles, 1970; Pauli and Cole, 1970). While skin temperature did not significantly deviate across timepoints, 5/6 aquanauts observed a reduction in skin temperature during saturation which is in line with the reduced metabolic rate driven by changes in cardiac, respiratory, temperature, and hemodynamic regulation. The most likely mechanism of reduced HR, respiratory rate, and skin temperature is an increase in vagal modulation. However, we cannot exclude possible alterations in cardiac efficiency and QT prolongation. Additionally, while evidence remains sparse, elevated PPN_2_ has been suggested to act as a beta-blocker, which may play a contributing role in the reduced HR at rest due to inherent elevations in PPN_2_ in AURL saturated environment. Lower respiratory rate may also be explained, in part, by the elevated airway resistance and respiratory muscle fatigue from inhaled gas density, but this has not been confirmed at atmospheric pressures < 6ATA (Dean and D’Agostino, 2008).

Increased parasympathetic and suppressed sympathetic modulation was confirmed by numerous metrics including time-domain (R–R intervals and RMSSD), frequency domain (nLF, HF, and LF/HF), Poincaré plot/non-linear (short-term autonomic regulation: SD1; long-term autonomic regulation: SD2), detrended fluctuation (DFA, α1 and DFA, α2) and composite metric (Stress and PNS Index) analyses. While LF was elevated, elevations in both LF and VLF are likely explained by increased global autonomic modulation in saturation, as LF controlled for total power (nLF) along with LF/HF were both decreased. These findings are in line with current evidence in acute HBS environment which have been shown to stimulate vagal modulation, reduced HR, and increased R–R interval variability and HF (Goyal et al., 2020). The increased vagal modulation in acute saturated environments has been attributed to both elevated O_2_ and increased pressure (independent of O_2_) (Lund et al., 1999). Mechanisms for this vagal responsiveness have been ascribed to vagal outflow compensating for O_2_ and/or pressure induced vasoconstriction (baroreflex), increased cardiac efficiency due to elevated PPO_2_ exposure in cardiac tissue, prolonged Q-T interval, and/or carotid chemoreflex inhibition (Demchenko et al., 2013; Paula-Ribeiro et al., 2019; Goyal et al., 2020).

Hemodynamic analyses revealed significant reductions in baseline cerebral blood flow velocity during saturation. Reductions in cerebral blood flow have also been observed in acute HBS (Omae et al., 1998) and hyperoxia in humans (Miller et al., 1970; Demchenko et al., 2000; Watson et al., 2000; Calvert et al., 2007). Reductions in cerebral blood flow are likely, in part, explained by the increased O_2_ availability in multiday HBS to cerebral and systemic tissues reducing tissue-driven blood flow demands. In support of this, Omae et al. (1998) found that the decreases in cerebral blood flow during 2 and 4 ATA were not due to increases in atmospheric pressure, but rather due to hyperoxemia. Our finding of reduced cerebral blood flow could also be explained by hypercapnia, but cerebral blood flow was also found reduced in hyperbaric O_2_ despite no change in arterial PCO_2_ (Omae et al., 1998). We did observe intact cerebral autoregulation as shown by the reduction in MAP but return of MCAv prior to the return of MAP during the prone-to-stand protocol. Although autoregulatory index was slightly reduced, the decrease in index was <1, which likely indicates a preserved cerebral autoregulatory mechanism, similar to previous studies examining autoregulation during hyperoxia (Nishimura et al., 2007; Ainslie et al., 2008). To our knowledge, cerebral autoregulation has not been investigated previously in hyperbaric conditions and the effects of higher gas pressures on the mechanism is unclear. We also observed a functional baroreflex response, primary mediator of cardiovascular and autonomic alterations to acute HBS exposure in model systems (Demchenko et al., 2013), as indicated by prone to standing reduced MAP and increased HR, similar to observations in acute saturation (Goyal et al., 2020).

Starting during early spaceflight (2–3 weeks) all the way to post spaceflight, HR and respiratory rate have been shown to progressively elevate over time (Xu et al., 2013), which is different from our observed depression and recovery of HR and respiratory rate during, and after multiday HBS, respectively. Body temperature has also been reported to be progressively elevated during spaceflight by up to 1°C (Stahn et al., 2017), another distinction from the 0.2°C reduction in skin temperature seen in the current study. Additionally, it has also been shown that various parameters of parasympathetic modulation decrease (SDRR, RMSSD, HF), while sympathetic parameters increase (LF/HF), during spaceflight at rest over similar exposure timeline to environmental stressors. Additionally, global autonomic modulation has been demonstrated to be repressed while α-DFA was unchanged in spaceflight, both different from what we observed in the multiday HBS detailed here. These observations in spaceflight (Fritsch-Yelle et al., 1994; Xu et al., 2013; Mandsager et al., 2015) are also in line with spaceflight simulation that investigated head-down bed rest, where parasympathetic modulation is consistently lowered (Crandall et al., 1994; Hughson et al., 1994; Sigaudo et al., 1996). One critical difference in physiologic stressors from spaceflight to the multiday HBS environment is gravitational impact on vascular flow and subsequent cardiac reactivity (baroreflex) (Fritsch-Yelle et al., 1994; Eckberg et al., 2010; Mandsager et al., 2015). We demonstrate that baroreflex sensitivity remains intact in the multiday HBS environment. Due to the critical nature of baroreflex across physiologic parameters, along with the critical nature of baroreflex ascribed to the autonomic regulation seen in acute HBS conditions (Demchenko et al., 2013), the altered hemodynamic function, subsequent baroreflex, and autonomic disruptions in microgravity provides the strongest rational for the inherent differences observed in multiday HBS versus the microgravitational extreme environment. Lastly, we confirmed the validity of the OURA ring device, which was able to accurately gather physiological recordings in accordance with the previously validated Polar V800 (HR and RMSSD; Supplementary Figure S1). Together, these findings demonstrate reduced HR, respiratory rate, skin temperature, and blood pressure likely explained by the intact baroreflex and global autonomic increase in parasympathetic and reduced sympathetic modulation in HBS. Additionally, our findings show reduced cerebral blood flow velocity and intact cerebral autoregulation, which may be explained by the increased O_2_ availability in HBS. These results demonstrate numerous similarities with acute HBS but are in stark contrast to adaptive responses observed in differential environmental extremes.

### Sleep Quantity and Quality

Aquanauts experienced increased trends in sleep latency and sleep quality (REM and deep sleep; Figure 5) during saturation. These changes in total sleep quantity and latency are likely explained by the increased stress of the mission HBS environments. However, the improvements in REM and deep sleep may be influenced by the increase in cardiac and autonomic recovery metrics (increased parasympathetic modulation, lower average and resting HR). These findings agree with observations made in sea level HBS chambers pressurized to simulate 20 m depth for 7 days where participants observed reduced total sleep quantity, increased sleep latency, and increased fatigue (Seo et al., 1999). However, subjects exposed to a prolonged duration of HBS for 60 days experienced increased total sleep and REM (Pauli and Cole, 1970; Buguet, 2007). We observed no changes in subjective sleep, as aquanauts reported moderately high sleep quality and alertness, as well as very mild pre-mission day fatigue, but with mild to moderate sleeplessness. This indicates that changes in objective sleep metrics do not necessarily parallel reported, subjective sleep evaluations. Sleep disturbances have been observed across various extreme environments (Buguet, 2007). Microgravitational space flight has been reported to reduce sleep duration and prolong sleep latency (Buguet, 2007; Barger et al., 2014), similar to our observations. However, subject sleep quality and alertness, as well as REM sleep have been reported to be reduced during spaceflight (Barger et al., 2014; Wu et al., 2018), illustrating differences between space flight and HBS.

### Body Composition

Aquanauts’ habituated to HBS for 9–10 days experienced weight loss (1.35 ± 0.23 kg) resulting from losses in systemic adipose tissue without detectable changes across muscle tissue metrics. While adipose tissue and skeletal muscle adaptations to multiday HBS have not been previously reported, bodyweight alone has been previously demonstrated to decrease by 2, 1.38, and 1.36 kg after 10, 45, and 60 days of saturation, respectively (Pauli and Clapper, 1967; Pauli and Cole, 1970; Zwart et al., 2009). Taken together, these four HBS studies suggest that the bodyweight adaptations likely occur within 10 days and subsequently stabilize. However, we cannot exclude the possibility for tissue composition change to progress for greater than 10 days. When comparing body composition changes in our analyses to other environmental extremes, the adaptive responses to bodyweight and fat-free mass appear to be similar across HBS, microgravity, and altitude for analyses of similar duration.

Acute and/or multiday body composition adaptations to other extreme environmental conditions have been previously described (Dunnwald et al., 2019; Laurens et al., 2019; Winnard et al., 2019). Microgravitational spaceflight or microgravity-modeled bed rest, has been shown to induce skeletal muscle and bone mineral density atrophy attributed to multiday mechanical underloading (disuse atrophy) (Laurens et al., 2019; Winnard et al., 2019). Observations across bed rest studies demonstrate modest effects on muscle function as early as 7 days. However, modest changes in muscle size (volume and cross sectional area) were not observed until 14 days (Winnard et al., 2019). Similarly, we also did not observe significant changes in muscle size within the less than a 14-day period of HBS exposure. Less investigation has been conducted on microgravity’s impact on adipose tissue because of the inherent abundance of such tissue. However, adipose tissue loss has been previously reported following space flight and bed-rest, where exercise (as a counter measure to combat muscle atrophy) was employed (Laurens et al., 2019). Of note, bed-rest participants allowed *ad libitum* food intake without exercise had unchanged fat mass (Blanc et al., 1998; Bergouignan et al., 2010). Conversely, bed-rest participants allowed *ad libitum* food intake with exercise had progressive adipose tissue atrophy (Bergouignan et al., 2010). Consequently, these prior studies suggest that adipose tissue loss in microgravity is driven by increased physical activity, not by microgravity itself. This demonstrates a clear discrepancy in tissue adaptation in the present observation of adipose tissue loss in HBS. Altitude has also been shown to induce adaptive changes in body composition (Dunnwald et al., 2019). A recent meta-analysis on altitude exposure details adaptive reductions in bodyweight, fat mass, and fat-free mass. However, like other environmental extremes, these tissue changes appear to be influenced by multiple factors including altitude level, length of exposure, physical activity, and alterations in nutritional status. In line with our findings, altitude exposure ≤ 10 days have commonly reported bodyweight and adipose tissue loss (Dunnwald et al., 2019), which appear progressive with prolonged exposure (Dunnwald et al., 2019; Laurens et al., 2019; Winnard et al., 2019). In disagreement with this hypothesis is the near identical weight loss observed in our study and longer HBS exposures (Pauli and Clapper, 1967; Pauli and Cole, 1970; Zwart et al., 2009). Together, these results demonstrate weight loss resulting from systemic adipose tissue loss without changes in muscle tissue.

## Limitations

While NASA NEEMO provides one of the only research environments to evaluate hyperbaric habituation over multiple days and is an ideal space flight mission analog replicating the mission demands, strict laboratory conditions across some study parameters (indicated in each section) were unrealistic due to NASA NEEMO ecological research environment and the prioritization of safe mission completion. Additionally, mission logistics and safety inherently limited participant *n* in data measurements, analyses, and at times, an ability to drawing meaningful statistical conclusions. Not all parameters had equal distribution of males and females, limiting extrapolation of findings across sexes. While NASA NEEMO 22 and 23 were analogous in hyperbaric exposure, mission design, and mission demands, minor difference in saturation length (NASA NEEMO 22: 10-day saturation; NASA NEEMO 23: 9-day saturation), EVA exposures, and daily mission tasks were observed. In some instances, individual data points and sub-cohort analyses could not be reported to ensure compliance with NASA LSAH privacy policy.

## Conclusion

Aquanauts living at HBS for 9–10 days during NASA NEEMO 22 and 23 experienced intrapersonal physical and mental burden, sustained good mood and work satisfaction, decreased HR and respiratory rates, increased parasympathetic and reduced sympathetic modulation, lower cerebral blood flow velocity, intact cerebral autoregulation and maintenance of baroreflex functionality, and losses in systemic bodyweight and adipose tissue. Together, these findings illustrate multiple novel insights into the unique human adaptation response to multiday HBS with clear overlap and distinction between other environmental extremes. Future studies should include the exploration of the molecular bases for these multi-component changes, and how these adaptations affect human response across other parameters of operational performance (Ari et al., 2020a,b,c).

## Data Availability Statement

Datasets presented in this article are not publicly available. NASA IRB protocol required the reporting of *mean* data sets, not individual subject data points. Requests to access the datasets should be directed to AK, akoutnik@ihmc.org or akoutnik@usf.edu.

## Ethics Statement

The studies involving human participants were reviewed and approved by Institute for Human and Machine Cognition, John Hopkins University, National Aeronautics and Space Agency, and/or European Space Agency IRB(s). The patients/participants provided their written informed consent to participate in this study.

## Author Contributions

AK, MF, CA, AP, JS, and DD led mission organization, development and implementation. All the authors were involved in mission organization, development and implementation, reviewed, revised, and approved final manuscript draft, agreed to accuracy and integrity of manuscript. AK led survey experimental design, sleep experimental design, sleep data processing, body composition data processing, and body composition experimental design and developed the original manuscript draft. AK and SM led survey data acquisition and processing. AK and MS-G led cardiorespiratory and autonomic experimental design and data acquisition. AK, MS-G, and BG led autonomic data processing. MF and JS led hemodynamic experimental design. MF, BS, and JS led hemodynamic data acquisition. MF, AK, and BS led hemodynamic data processing. AK, SM, and MM led sleep data acquisition. AK, KN, SM, JD, and CR led body composition data acquisition. AK, MF, and MS-G led statistical analyses. All authors contributed to the article and approved the submitted version.

## Conflict of Interest

The authors declare that this study received funding from Ketone Technologies LLC. Ketone Technologies LLC is an entity which supports research on extreme environments for which CA and DD serve as CEO and CSO, respectively. CA and DD received no personal compensation for these studies and were involved in project development, logistical implementation, and final manuscript development. These interests have been reviewed and managed by NASA in accordance with its Institutional and Individual Conflict of Interest policies. The remaining authors declare that the research was conducted in the absence of any commercial or financial relationships that could be construed as a potential conflict of interest.

## Abbreviations

ATA: Atmospheres Absolute/Barometric Pressure
AURL: Aquarius Undersea Research Laboratory
BMD: bone mineral density
BMI: body mass index
CO_2_: carbon dioxide
DFA α 1: short-term detrended fluctuation analysis
DFA α 2: long-term detrended fluctuation analysis
DBP: diastolic blood pressure
EVAs: extravehicular activities
HBS: hyperbaric and/or saturated
HF: high-frequency
HR: heart rate
JSC: NASA Johnson Space Center
LF: low frequency
MAP: mean arterial pressure
MCAv: middle cerebral artery flow velocity
MCAv%: middle cerebral artery velocity percent change
NASA: National Aeronautic Space Agency
NEEMO: National Aeronautic Space Agency Extreme Environment Mission Operation
N_2_: nitrogen
Nu: normalized units
nLF: normalized low frequency
O_2_: oxygen
PP: partial pressure
PNS: parasympathetic nervous system
REM: rapid eye movement
RMSSD: Root Mean Square of Successive Differences
RNOS: reactive nitrogen and oxygen species
RR Interval: Mean R–R Interval
SBP: systolic blood pressure
SD1: Poincaré
Perpendicular: Standard Deviation
SD2: Poincaré
Parallel: Standard Deviation
SNS: sympathetic nervous system
VLF: very low frequency.
