# Supplementary material for: Human Adaptations to Multiday Saturation on NASA NEEMO
Source: Front Physiol. 2021 Jan 12;11:610000. doi: 10.3389/fphys.2020.610000 (PMC7835980; doi:10.3389/fphys.2020.610000)
Supplement: Supplementary file 1 [file Table_1.docx]

**Supplemental Data**

**Table S1. Variable. Aquanaut Raw Mean Autonomic Values.** Raw means reported for pre-saturation, saturation, and post-saturation timepoints for all logarithmically adjusted parameters which were not normally distributed values. *N*=7 (male, *n*=5; female, *n*=2). **Data:** Mean ± SD. Abbreviations: RR Interval, Mean R-R Interval; HF, High Frequency Power Domain.

| **Aquanaut Raw Mean Autonomic Values** | | | |
| --- | --- | --- | --- |
| **Timepoint** | **Pre-Saturation** | **Saturation** | **Post-Saturation** |
| **RR Interval** | 884.5±101.4 | 976.8±130.3 | 877.9±81.4 |
| **HF** | 427.9±580.5 | 1143.0±1145.2 | 338.2±301.8 |
| **Stress Index** | 8.7±3.4 | 6.6±3.4 | 8.7±3.4 |

**S.1 OURA and Polar Correlation Analyses**

Polar and OURA devices demonstrated overlapping directional change and significant correlations across heart rate (r=0.72, p<0.0001) and RMSSD (r=0.98, p<0.0001), illustrating that OURA ring technology can provide valid assessment of cardiac and autonomic function (Figure S1).

**Supplemental Figure 1. Polar and OURA Correlation Analyses.** **(A)** Polar heart rate **(B)** OURA heart rate **(C)** Polar root mean square of successive difference (RMSSD), and **(D)** OURA RMSSD were measured across overlapping time periods. **(E)** Heart Rate (HR) and **(F)** RMMSD were assessed across devices on all overlapping timepoints across all subjects to determine Pearson r correlations between parameters. N= A,C: *N*=7 (male, *n*=5; female, *n*=2). B, D *N*=6 (male, *n*=6); E-F: *N*=30 (male, *n*=6 across all recorded timepoints). **Data:** Mean ± SD. Abbreviations: RMSSD, Root Mean Square of Successive Differences; HR, Heart Rate; **p<0.05, **p<0.01*. Raw p-values are reported for non-significant changes where p≤0.100*.*
